# Sex-Related Differences in the Effects of the Mediterranean Diet on Glucose and Insulin Homeostasis

**DOI:** 10.1155/2014/424130

**Published:** 2014-10-09

**Authors:** Alexandra Bédard, Louise Corneau, Benoît Lamarche, Sylvie Dodin, Simone Lemieux

**Affiliations:** ^1^Institute of Nutrition and Functional Foods (INAF), Laval University, 2440 Hochelaga Boulevard, Québec, QC, Canada G1V 0A6; ^2^Department of Food Science and Nutrition, Pavillon Paul-Comtois, Laval University, 2425 rue de l'Agriculture, Québec, QC, Canada G1V 0A6; ^3^Department of Obstetrics and Gynaecology, Pavillon Ferdinand-Vandry, Laval University, 1050 Medicine Avenue, Québec, QC, Canada G1V 0A6

## Abstract

*Objective*. To document sex differences in the impact of the Mediterranean diet (MedDiet) on glucose/insulin homeostasis and to verify whether these sex-related effects were associated with changes in nonesterified fatty acids (NEFA). *Methods*. All foods were provided to 38 men and 32 premenopausal women (24–53 y) during 4 weeks. Variables were measured during a 180 min OGTT before and after the MedDiet. *Results*. A sex-by-time interaction for plasma insulin iAUC was found (men: −17.8%, *P* = 0.02; women: +9.4%, *P* = 0.63; *P* for sex-by-time interaction = 0.005). A sex-by-time interaction was also observed for insulin sensitivity (Cederholm index, *P* = 0.03), for which only men experienced improvements (men: +8.1%, *P* = 0.047; women: −5.9%, *P* = 0.94). No sex difference was observed for glucose and C-peptide responses. Trends toward a decrease in NEFA AUC (*P* = 0.06) and an increase in NEFA suppression rate (*P* = 0.06) were noted, with no sex difference. Changes in NEFA were not associated with change in insulin sensitivity. *Conclusions*. Results suggest that the more favorable changes in glucose/insulin homeostasis observed in men compared to women in response to the MedDiet are not explained by sex differences in NEFA response. This clinical trial is registered with clinicaltrials.gov NCT01293344.

## 1. Introduction

Diabetes has reached epidemic proportion with 382 million people worldwide affected by this disease [1]. An important sex difference has been highlighted in the health consequences of type 2 diabetes mellitus with a 50% higher risk of coronary heart disease mortality in diabetic women compared to diabetic men [2]. In addition to this higher mortality risk found in diabetic women, there is growing evidence that the response to early lifestyle interventions aiming at counteracting insulin resistance and thereby reducing the risk of developing diabetes and its cardiovascular complications may be sex-specific, with women having generally fewer benefits than men [3]. In fact, greater improvements in insulin sensitivity have been observed in men compared to women in response to exercise training interventions [4, 5]. Moreover, it has been suggested that body weight loss improves insulin sensitivity to a greater extent in men [6]. Very few studies have yet documented potential sex-related differences in response to the adoption of healthy dietary habits. Moreover, the few studies that have examined this issue were generally achieved in uncontrolled nutritional contexts and gender differences in the adherence to prescribed dietary habits in these studies may have confounded the interpretation of results obtained.

The adoption of the traditional Mediterranean diet (MedDiet) is now widely recommended for its various health benefits [7]. Among others, the adoption of this dietary pattern has been identified as a useful approach to improve glycemic control and prevent the development of insulin resistance and type 2 diabetes mellitus [8, 9]. However, little is known about how the MedDiet leads to improvements in glucose and insulin homeostasis. One possible mechanism is a decrease in nonesterified fatty acid (NEFA) concentration in response to the MedDiet. In fact, from a metabolic standpoint, elevated NEFA concentration is established as one of the main contributing factors for the development of insulin resistance through the impairment of both hepatic glucose production and insulin action in peripheral tissues [10].

Using a fully controlled nutritional context, the aim of this study was therefore to examine how sex modifies the short-term impact of a MedDiet on variables related to glucose and insulin homeostasis. We also verified if changes in glucose and insulin homeostasis were related to changes in NEFA concentration in men and women.

## 2. Subjects and Methods

### 2.1. Subjects

Thirty-eight men and 32 premenopausal women aged from 24 to 53 years were recruited. In addition to having slightly elevated plasma low-density lipoprotein- (LDL-) cholesterol concentration (between 3.4 and 4.9 mmol/L) or a total cholesterol to high-density lipoprotein- (HDL-) cholesterol ratio ≥5.0, participants had at least one of the four following cardiovascular risk factors: (1) waist circumference > 94 cm in men and >80 cm in women [12]; (2) triacylglycerol (TAG) > 1.7 mmol/L; (3) fasting glycemia between 6.1 and 6.9 mmol/L; and (4) blood pressure concentrations ≥ 130/85 mm Hg. Exclusion criteria included a significant weight change (>2.5 kg) in the three months before the study, cardiovascular events, prior diagnosis of type 2 diabetes mellitus, use of medication that could affect dependent variables under study (namely, lipid-lowering, hypoglycemic, insulin sensitizers and antihypertensive medications), smoking, pregnancy, and use of systemic hormonal contraceptives. Premenopausal status was determined by a regular menstrual cycle for the last three months and a FSH measurement <20 IU/L during the early follicular phase. Power analyses for repeated measures and within-between interactions showed that a total sample size of *n* = 68 is sufficient to detect significant differences in the outcomes with a small effect-size estimate (Cohen's *d* of 0.15 [13]) and with an *α* = 0.05 and a power (1 − *β* error probability) of 0.95 (G*Power Version 3.0.10, Franz Faul, Universität Kiel, Germany). The present study was conducted according to the guidelines laid down in the Declaration of Helsinki (1964). All procedures involving human subjects were approved by the Laval University Research Ethics Committee on human experimentation. Written informed consent was obtained from all subjects. This clinical trial was registered at clinicaltrials.gov as NCT01293344.

### 2.2. Study Design

The design and methods of this study were previously described in detail [11]. Firstly, during a 4-week run-in period, participants had to comply with the recommendations of Canada's Food Guide [14] as prescribed by a registered dietician. Canada's Food Guide is an educational tool which promotes a healthy eating pattern in order to reduce the risk of many chronic diseases and to achieve overall health and vitality. Participants were also instructed to maintain constant their body weight and physical activity participation. The purpose of this run-in period was to ensure similar dietary habits between men and women prior to the controlled MedDiet phase, a goal which has been achieved as reported in a previous publication [11].

The 4-week controlled MedDiet followed immediately the run-in period. All foods and drinks were provided for participants according to a 7-day cyclic menu. It has been shown that 4 weeks of nutritional intervention under controlled conditions is sufficient to obtain significant changes in glucose/insulin homeostasis [15–18]. Details about the composition of the MedDiet have been previously reported [11]. The percentages of energy derived from lipids, carbohydrates, proteins, and alcohol were, respectively, of 32%, 46%, 17%, and 5%. Participants were instructed to consume only foods and drinks provided and their entire meals. Compliance of participants was evaluated with a checklist throughout the controlled MedDiet phase. Participants were also instructed to maintain their usual physical activity.

The controlled MedDiet phase aimed at being isocaloric in order to control for the potential confounding effect of body weight change. On each weekday, body weight was measured immediately before lunch and foods and energy provided were revised if necessary to minimize body weight fluctuations. In women, all tests were carried out in the early follicular phase of their menstrual cycle (from the third to ninth day of the menstrual cycle; mean duration of the feeding period in women is 28.8 ± 4.3 days) since fluctuations in female hormones may influence glucose and insulin homeostasis [19].

### 2.3. Laboratory Measurements

After a 12-hour overnight fast, basic lipid profile was measured after the run-in period (i.e., just before the controlled MedDiet phase). Serum cholesterol, HDL-cholesterol, and TAG concentrations were measured using commercial reagents on a Modular P chemistry analyzer (Roche Diagnostics, Mannheim, Germany). LDL-cholesterol was obtained by the equation of Friedewald et al. [20]. Moreover, a 180 min oral glucose tolerance test (OGTT) with 75 g of glucose in solution was performed just before and after the controlled MedDiet. Blood samples were collected into vacutainer tubes containing ethylene diamine tetra-acetic acid (EDTA) at −15, 0, 15, 30, 45, 60, 90, 120, 150, and 180 min. At each time, plasma glucose concentration was determined using the hexokinase-glucose-6-phosphate dehydrogenase method [21], plasma insulin concentration was measured by radioimmunoassay [22], plasma C-peptide concentration was measured by a modification of the method of Heding [23] with polyclonal antibody A-4741 from Ventrex (Portland, ME, USA) and polyethylene glycol precipitation [22], and plasma NEFA concentration was assessed with an enzymatic detection kit (ZenBio, Research Triangle Park, NC, USA). Fasting values were computed as the mean of −15 and 0 min values. Incremental areas under the curve (iAUC) for glucose, insulin, and C-peptide and the area under the curve (AUC) for NEFA were calculated for the 180 min interval using the trapezoidal method.

The incremental peak was calculated as the difference between the concentration of glucose, insulin, and C-peptide at peak and the concentration in the fasting state during the OGTT. The percentage of suppression of NEFA was calculated in each participant using this formula: [(fasting NEFA − the lowest NEFA concentration during the OGTT)/fasting NEFA] × 100. Hepatic insulin sensitivity was assessed by the homeostasis model assessment (HOMA-IS) approach index (1/[fasting glucose (mmol/L) × fasting insulin (mIU/L)/22.5]). Peripheral insulin sensitivity was assessed with the Cederholm index according to the following formula: [75 000 + (fasting glucose − glucose 120 min postload) × 1.15 × 180 × 0.19 × body weight]/[120 × log (mean insulin) × mean glucose] in which glucose concentrations are expressed in mmol/L, insulin concentrations in mIU/L, and body weight in kg [24].

As proposed by the Canadian Diabetes Association [25], normal glucose tolerance was defined as a fasting plasma glucose <6.1 mmol/L and plasma glucose 120 min postload <7.8 mmol/L, isolated impaired fasting glucose as fasting glucose between 6.1 and 6.9 and glucose 120 min postload <7.8 mmol/L, isolated impaired glucose tolerance as fasting glucose <6.1 mmol/L and glucose 120 min postload between 7.8 and 11.0 mmol/L, and both impaired fasting glucose and impaired glucose tolerance as fasting glucose between 6.1 and 6.9 and glucose 120 min postload between 7.8 and 11.0 mmol/L and type 2 diabetes mellitus was defined as fasting glucose ≥7.0 mmol/L or glucose 120 min postload ≥11.1 mmol/L.

### 2.4. Anthropometric and Blood Pressure Measurements

After the run-in period (i.e., just before the controlled MedDiet phase), body weight, height, and waist circumference measurements were performed using standardized methods [26]. Systolic and diastolic blood pressure levels were measured on the right arm using an automated blood pressure monitor (BPM 300-BpTRU: Vital Signs Monitor) as previously described [11].

### 2.5. Statistical Analyses

Data were collected after the run-in period (i.e., just before the controlled MedDiet phase) and immediately after the controlled MedDiet. For variables not normally distributed, a transformation was performed. To determine differences between men and women for characteristics before the controlled MedDiet phase, Student's *t*-test for unpaired data was used. Time and sex-by-time interaction effects on dependent variables were assessed by using MIXED procedures for repeated measurements followed by Tukey-Kramer tests. Statistical analyses were adjusted for values before the MedDiet phase when a difference between men and women was observed. Associations between variables were assessed by Pearson's correlation analyses. Although the controlled MedDiet phase aimed at being isocaloric, both men and women had a small but significant weight loss (−1.2 kg or 1.3% of initial body weight in men and −0.5 kg or 0.7% in women). However, no significant change was observed in both men and women for waist circumference (−0.3 cm or 0.3% of initial waist circumference in men and −0.8 cm or 0.8% in women). All results are adjusted for this small change in body weight during the controlled MedDiet phase. We excluded one man from our analyses due to illness, which led to a significant reduction in food intake during several days at the end of the controlled MedDiet phase. Therefore, 37 men and 32 women were included in the analyses. A *P* ≤ 0.05 (two-sided) was considered as statistically significant. All analyses were performed with the SAS statistical package version 9.2 (SAS Institute Inc., Cary, NC, USA).

## 3. Results 

### 3.1. Participant Characteristics after the Run-In Period

As previously reported in another publication [11], men and women had similar age and BMI (Table 1). Waist circumference measurements were higher in men than in women. On average, both men and women were characterized by a slightly deteriorated lipid profile and normal blood pressure levels, but some sex differences were noted for these metabolic variables. Specifically, men displayed higher values for TAG and systolic and diastolic blood pressure and a lower value for HDL-cholesterol concentration than women. For variables related to glucose and insulin homeostasis, men and women had comparable values before the controlled MedDiet, except for fasting glucose, for which men had a higher value than women. There was no sex difference for C-peptide and NEFA concentrations in the fasting state.

Forty-one participants had a normal glucose tolerance (20 men and 21 women), 10 had an isolated impaired fasting glucose (nine men and one woman), nine were characterized by an isolated impaired glucose tolerance (five men and four women), and four participants had both an impaired fasting glucose and an impaired glucose tolerance (two men and two women). Even if none of the participants had received a diagnosis of type 2 diabetes mellitus prior to their inclusion in this study, one man and four women met diagnosis criteria for type 2 diabetes mellitus.

### 3.2. Glucose, Insulin, and C-Peptide Responses to the MedDiet

**Figure 1:**
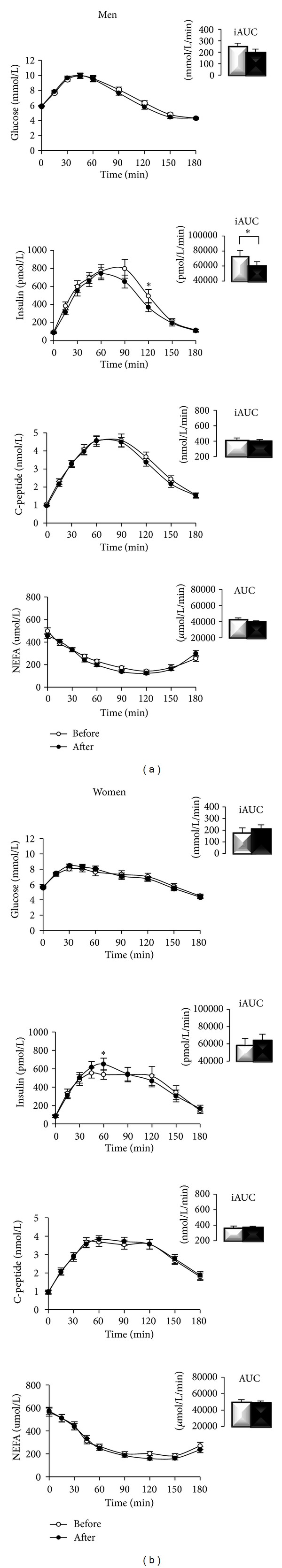
Glucose, insulin, C-peptide, and nonesterified fatty acid (NEFA) concentrations observed in men (left, *n* = 37) and women (right, *n* = 32) before (–o–) and after (–●–) the 4-week controlled Mediterranean diet. Analyses were adjusted for values before the controlled phase and body weight change during the controlled phase. MIXED procedures for repeated measurements followed by Tukey-Kramer tests were used. Sex-by-time interaction effects were found for insulin iAUC (*P* = 0.005) as well as for insulin 60 min postload (*P* = 0.02) and insulin 120 min postload (*P* = 0.03). *Decreases in insulin iAUC and insulin 120 min postload were found only in men (resp., *P* = 0.02 and *P* = 0.03) whereas only women experienced an increase in insulin 60 min postload (*P* = 0.04).

There was no sex-by-time interaction or time effect for fasting glucose, insulin, and C-peptide concentrations (Figure 1). During the 75 g OGTT, a sex-by-time interaction for plasma insulin iAUC was found, with men having a significant decrease whereas women experienced a nonsignificant increase (men −17.8%, *P* = 0.02, women +9.4%, *P* = 0.63, *P* for sex-by-time interaction = 0.005) (Figure 1). More precisely, when statistical analyses were performed at each time point of the OGTT, sex-by-time interaction effects were found at 60 and 120 min (resp., *P* = 0.02 and *P* = 0.03). Specifically, a significant increase in insulin concentrations at 60 min was observed in women (+21.1%, *P* = 0.04) whereas men showed a nonsignificant decrease (−3.4%, *P* = 0.95). For insulin concentration at 120 min, men showed a greater reduction after MedDiet than women (−25.4% in men, *P* = 0.03 and −10.3% in women, *P* = 0.99). There was no sex-by-time interaction or time effect for glucose and C-peptide iAUCs (Figure 1) as well as for incremental peaks of glucose, insulin, and C-peptide in response to the MedDiet (Table 2).

### 3.3. Insulin Sensitivity Response to the MedDiet

No time or sex-by-time interaction effect was observed for hepatic insulin sensitivity as calculated with the HOMA-IS index (Table 2). However, a sex-by-time interaction was found for peripheral insulin sensitivity as calculated by the Cederholm index (*P* = 0.03), with men showing an increase (+8.1%, *P* = 0.047) whereas a nonsignificant decrease was observed in women (−5.9%, *P* = 0.94) (Table 2).

### 3.4. NEFA Response to the MedDiet

No time or sex-by-time interaction was observed for fasting NEFA concentration. A trend toward a decrease in NEFA AUC was noted in men and women combined (−6.4%; time effect: *P* = 0.06) but no sex-by-time interaction was observed for this variable (Figure 1). Moreover, a tendency for an increase was found for the NEFA suppression rate when the whole sample was considered (+3.5%; time effect: *P* = 0.06) (Table 2), but no sex-by-time interaction effect was noted.

### 3.5. Associations between Changes in NEFA and Changes in Glucose and Insulin Homeostasis in Response to the MedDiet

In men, changes in fasting NEFA concentration in response to the MedDiet were positively associated with concurrent variations in the incremental peak of glucose and glucose iAUC and negatively associated with changes in the peripheral insulin sensitivity as measured by the Cederholm index (Table 3). In women, changes in fasting NEFA concentration and NEFA AUC with MedDiet were negatively associated with concurrent variations in fasting C-peptide concentration. Moreover, changes in NEFA AUC were positively associated with variations in glucose concentration at 120 min and glucose iAUC in women. Finally, changes in NEFA suppression rate were positively associated with variations in the incremental peak of glucose in men and negatively associated with variations in fasting glucose concentration in women.

## 4. Discussion

The present study reports for the first time, in the context of a fully controlled nutritional feeding protocol, detailed sex-related differences in glucose and insulin homeostasis in response to consumption of a traditional MedDiet, a healthy food pattern recognized to prevent the development of insulin resistance and type 2 diabetes mellitus [8, 9]. Specifically, our results showed that, in men, MedDiet improves peripheral insulin sensitivity as measured by the Cederholm index and reduces insulin iAUC during an oral glucose challenge. In contrast, women had no beneficial change for these variables in response to the MedDiet. Sex differences in insulin homeostasis found in this study are not explained by sex differences in NEFA response to the MedDiet. However, sex-specific pattern of associations between changes in variables related to glucose and insulin homeostasis and changes in NEFA concentrations were found.

The beneficial effects of the MedDiet on peripheral insulin sensitivity in men are in concordance with the previous literature. In fact, many characteristics of the MedDiet, such as its high content in monounsaturated fatty acids, fibers, and polyphenolic compounds and the moderate alcohol intake habitually found with this dietary pattern, have been previously identified as improving insulin sensitivity through several mechanisms and independently of weight loss, as previously reviewed [27]. However, our results add to the previous literature since we showed that, when considered as a whole, the MedDiet leads to beneficial effects on insulin homeostasis, but these benefits seem to be sex-specific, with men having more favorable effects than women.

Improvements in peripheral insulin sensitivity observed in men after the MedDiet suggest a higher effectiveness of insulin-stimulated glucose uptake in peripheral tissues, leading to a decrease in insulin needs. This decrease in insulin needs can in turn explain the decrease in insulin concentrations observed in men during the OGTT. Two mechanisms can explain this decrease in insulin concentrations with MedDiet: reduced insulin secretion or enhanced insulin clearance from the circulation. Since C-peptide concentration has been identified as an indirect indicator of in vivo pancreatic beta-cell insulin secretion [28] and no significant change in C-peptide concentration occurred in this study, our results suggest that reduced insulin concentrations in men in response to the MedDiet are likely to be primarily due to enhanced insulin clearance. Insulin clearance was not measured in the present study and further work is warranted to confirm this hypothesis.

Elevated circulating NEFA concentrations have been identified as one of the main contributing metabolic factors for the development of insulin resistance. More precisely, NEFA induce insulin resistance through activation of a serine-kinase cascade, leading to the inhibition of the insulin signaling pathway in adipose tissue, skeletal muscle, and liver [29]. Results from the present study suggest that sex differences in insulin homeostasis are not due to sex differences in NEFA response to the MedDiet. Other potential factors related to insulin resistance may explain these sex differences in the response to MedDiet. Among others, it has been demonstrated that estrogen favors insulin sensitivity, via its numerous beneficial effects on insulin and glucose homeostasis, adipose tissue distribution, oxidative stress, and inflammatory status [30]. Since a decrease in estrogen levels has been previously highlighted in response to the MedDiet in women [31], we can speculate that a potential decrease in estrogen levels may have counteracted the beneficial effects of the MedDiet in premenopausal women from our study.

The lack of a significant reduction in glucose response to the MedDiet in men, despite improvements in insulin homeostasis, may be explained by the fact that the majority of our subjects had a normal glucose tolerance before the controlled MedDiet phase. In fact, previous studies have highlighted that the MedDiet leads to beneficial effects on glucose concentrations in individuals with type 2 diabetes mellitus [8]; however, the impact of the MedDiet in individuals without type 2 diabetes is still unknown [32–37]. In a weight loss context, Shai and collaborators have observed decreases in both glucose and insulin concentrations in individuals characterized by type 2 diabetes mellitus with the adoption of the MedDiet whereas only reduced insulin concentrations were found in nondiabetic individuals [32]. Given the limited number of our participants who met diagnosis criteria for type 2 diabetes mellitus, we cannot verify this issue in the present study.

Sex differences in the pattern of associations between variables related to glucose and insulin homeostasis and NEFA concentrations have been reported in this study in response to the MedDiet. Our results showed that changes in fasting NEFA concentrations after the MedDiet are negatively associated with parallel changes in peripheral insulin sensitivity in men, meaning that men who decreased the most their fasting NEFA in response to the MedDiet had greater improvements in peripheral insulin sensitivity. This association was not observed in women.

Since women characterized by type 2 diabetes mellitus are at higher risk of coronary heart disease mortality than diabetic men [2], sex differences highlighted in this study have therefore important clinical implications. Indeed, women have been underrepresented in the majority of clinical trials; therefore we know little about efficient strategies aiming at improving glucose and insulin homeostasis in women. Thus our results, along with those from other studies, highlight the importance of including both men and women in future studies in order to identify effective interventions for women in prevention of diabetes and its cardiovascular-related risk. However, despite these results, it is important to stress out the fact that the adoption of the MedDiet brings beneficial effects on the cardiovascular health with regard to the lipid profile and blood pressure levels in both men and women, with no sex difference for these cardiovascular risk factors [11], suggesting beneficial cardiovascular effects of the MedDiet, regardless of the sex.

A major strength of this study is its strictly controlled design during the MedDiet phase, ensuring optimal control over energy intake and diet quality. One limitation is that peripheral insulin sensitivity was assessed using the Cederholm index, a surrogate marker, rather than the gold-standard euglycemic-hyperinsulinemic clamp. However, the Cederholm index has been demonstrated to correlate closely with specific measures of insulin sensitivity derived from the euglycemic-hyperinsulinemic clamp [38, 39]. Another limitation is the heterogeneity of the study group, consisting of subjects with various degrees of glucose tolerance and insulin sensitivity. However, for markers related to glucose and insulin homeostasis, except for fasting glucose, men and women had comparable values before the controlled MedDiet, which facilitates the comparison between sexes.

## 5. Conclusions

In summary, short-term consumption of a traditional MedDiet leads to beneficial effects on insulin homeostasis in men but not in premenopausal women. Sex differences in insulin homeostasis are not due to sex differences in NEFA response to the MedDiet. Since hyperinsulinemia increases the risk of cardiovascular disease and type 2 diabetes mellitus [40], our results suggest that the MedDiet may have particular benefits for cardiovascular health in men through its favorable effects on postprandial insulin concentrations. Further studies are needed to document underlying mechanisms behind this sex difference in response to the MedDiet.

## Figures and Tables

**Table 1:** Characteristics of men and women after the run-in period.

	Men (*n* = 37)		Women (*n* = 32)		Sex difference
	Mean	SD	Mean	SD	*P* value
Age (years)^a^	42.6	7.3	41.2	7.3	0.43
Body weight (kg)^a,b^	91.8	14.0	78.0	14.7	**<0.0001**
BMI (kg/m^2^)^a,b^	29.2	3.2	29.6	5.4	0.97
Waist circumference (cm)^a,b^	102.6	10.7	96.4	10.5	**0.01**
TAG (mmol/L)^a,b^	1.86	1.17	1.36	0.63	**0.03**
Total cholesterol (mmol/L)^a^	5.56	0.91	5.40	0.60	0.38
LDL cholesterol (mmol/L)^a^	3.61	0.72	3.47	0.52	0.36
HDL cholesterol (mmol/L)^a,b^	1.09	0.31	1.30	0.26	**0.002**
Systolic blood pressure (mm Hg)^a^	117.1	12.6	108.6	10.4	**0.004**
Diastolic blood pressure (mm Hg)^a^	80.3	9.0	73.5	9.0	**0.003**
Fasting glucose (mmol/L)^a,b^	5.89	0.37	5.68	0.63	**0.04**
Glucose 120 min postload (mmol/L)^a^	6.36	1.81	7.02	2.48	0.21
Fasting insulin (pmol/L)^a,b^	96.0	57.6	86.2	79.3	0.19
Insulin 120 min postload (pmol/L)^a,b^	493.8	443.0	520.9	588.7	0.84
Fasting C-peptide (nmol/L)^b^	1.04	0.37	0.94	0.53	0.21
Fasting NEFA (umol/L)^b^	497.8	180.8	563.8	199.7	0.15

SD, standard deviation; BMI, body mass index; TAG, triacylglycerol; LDL, low-density lipoprotein; HDL, high-density lipoprotein; NEFA, nonesterified fatty acids.

These characteristics were measured after the run-in period that is immediately before the controlled MedDiet phase. Differences between men and women were assessed by Student's *t*-test for unpaired data.

^a^These characteristics have been reported in a previous publication [11].

^b^Analysis was performed on transformed values.

**Table 2:** Glucose and insulin homeostasis response to the 4-week controlled Mediterranean diet.

	Men (*n* = 37)				Women (*n* = 32)				Time	Sex-by-time interaction
	Before	SEM	After	SEM	Before	SEM	After	SEM	*P* value	
Incremental peak										
Glucose (mmol/L)^a,b^	4.66	0.25	4.66	0.26	3.35	0.30	3.53	0.28	0.36	0.32
Insulin (pmol/L)^a^	864	95	787	69	715	99	688	77	0.52	0.57
C-peptide (nmol/L)^b,c^	4.33	0.31	4.14	0.24	3.47	0.24	3.35	0.17	0.37	0.87
HOMA-IS index^a,d^	0.408	0.105	0.356	0.041	0.606	0.172	0.496	0.071	0.55	0.68
Cederholm index^a^	20.9	1.1	22.6	0.9^e^	24.6	1.5	23.1	1.2	0.18	**0.03**
NEFA suppression rate (%)	73.3	2.0	74.1	2.3	70.5	2.1	75.1	2.0	0.06	0.18

SEM, standard error of the mean; HOMA-IS index, homeostasis model assessment for insulin sensitivity index; NEFA, nonesterified fatty acids.

Time and sex-by-time interaction effects on dependent variables were assessed by using MIXED procedures for repeated measurements followed by Tukey-Kramer tests.

^a^Analysis was performed on transformed values.

^b^Differences between men and women were observed with Student's t-test before the controlled MedDiet phase; P = 0.0004 for the incremental peak of glucose and P = 0.04 for the incremental peak of C-peptide. For these variables, statistical analyses were adjusted for values before the controlled MedDiet phase.

^c^For women, n = 31 for the incremental peak of C-peptide due to a missing value.

^d^Results about HOMA-IS have been reported in a previous publication [11].

^e^A significant decrease was observed for the Cederholm index in men, P = 0.047.

**Table 3:** Pearson's correlation coefficients for the associations between changes in nonesterified fatty acids (NEFA) concentrations and changes in variables related to the glucose and insulin homeostasis in men and women in response to the Mediterranean diet.

	Men			Women		
	Fasting NEFA	NEFA AUC	NEFA suppression rate	Fasting NEFA	NEFA AUC	NEFA suppression rate
	*r*	*r*	*r*	*r*	*r*	*r*
Glucose						
Fasting	0.11	−0.15	0.25	−0.32	−0.19	−0.36*
Peak	0.46**	0.24	0.36*	−0.11	0.07	−0.01
120 min	0.23	0.07	0.08	0.02	0.42*	−0.15
iAUC	0.42*	0.23	0.26	0.31	0.44*	0.21
Insulin						
Fasting	0.12	0.07	0.12	−0.32	−0.37	−0.06
Peak	0.14	0.07	0.16	0.12	−0.09	0.08
120 min	0.11	0.02	0.05	0.08	−0.07	0.22
iAUC	0.23	0.18	0.27	0.22	0.08	0.24
C-peptide						
Fasting	0.03	−0.14	0.18	−0.45*	−0.41*	−0.30
Peak	0.05	0.14	0.06	0.01	0.16	−0.09
120 min	−0.02	0.10	0.01	0.09	0.08	0.06
iAUC	0.08	0.15	0.09	0.16	0.28	−0.02
HOMA-IS index	−0.07	0.10	−0.11	0.10	0.15	−0.02
Cederholm index	−0.39*	−0.18	−0.29	0.15	−0.12	−0.07

*P < 0.05, **P < 0.01.

NEFA, nonesterified fatty acids; AUC, area under the curve; iAUC, incremental area under the curve; HOMA-IS index, homeostasis model assessment index for insulin sensitivity.

*n* = 37 for men, except for associations including incremental peaks of glucose, insulin and C-peptide, iAUC for glucose, insulin, C-peptide and NEFA, fasting C-peptide, C-peptide 120 min postload, and the Cederholm index for which *n* = 35 due to missing values.

*n* = 32 for women, except for associations including peaks of glucose (*n* = 31), insulin (*n* = 31) and C-peptide (*n* = 30), iAUC for glucose (*n* = 29), insulin (*n* = 29), C-peptide (*n* = 28) and NEFA (*n* = 28, except for associations with variables related to C-peptide, *n* = 27), fasting C-peptide (*n* = 31), C-peptide 120 min postload (*n* = 31), and the Cederholm index (*n* = 29) due to missing values.
